# Influence of socioeconomic status on physical and psychological wellbeing during the cancer care continuum: a longitudinal and logistic regression analysis of cancer patients in New Delhi, India

**DOI:** 10.1007/s00520-026-10734-7

**Published:** 2026-05-07

**Authors:** Ishaan Rahman, Mevhibe Hocaoglu

**Affiliations:** 1 Faculty of Life Sciences & Medicine, King’s College London, London, UK; 2 Florence Nightingale Faculty of Nursing, Midwifery & Palliative Care, King’s College London, London, UK

**Keywords:** Cancer symptom burden, Health disparities, India, Psycho-oncology, Socioeconomic status, Supportive care

## Abstract

This study examined the influence of socioeconomic status (SES) on physical, psychological, and wellbeing symptom burden among palliative cancer patients in New Delhi, India, to identify manifestations of inequity across the cancer care continuum. A secondary analysis of a prospective cohort dataset of 240 cancer patients receiving palliative care was conducted. Participants completed a baseline survey (T1); 120 were followed up after 1 month (T2). Symptom burden was measured using Likert scales for physical, psychological, and wellbeing outcomes. Chi-square tests assessed associations between SES and symptom severity. Ordinal logistic regressions examined the predictive effects of education, income, and slum residence, adjusting for age, sex, and cancer stage. Low education and financial insecurity were associated with higher symptom burden. At T1, patients with no formal education reported more severe weakness than those with tertiary education (OR = 2.84, 95% CI 1.25–6.42, *p* = 0.012). Not coping on income was associated with greater weakness (OR = 2.67, 95% CI 1.48–4.82, *p* = 0.001) and depression (OR = 1.94, 95% CI 1.10–3.40, *p* = 0.021). At T2, this was also associated with more pain (OR = 2.44, 95% CI 1.02–5.81, *p* = 0.044) and feeling less informed (OR = 2.58, 95% CI 1.03–6.44, *p* = 0.043). Slum residence was associated with higher odds of depression (OR = 0.25, 95% CI 0.09–0.70, *p* = 0.008). SES strongly shaped symptom burden, with low-income, less-educated, and slum-resident patients disproportionately affected. Findings highlight the need for cancer care strategies integrating financial protection, patient education, and mental health services to mitigate inequities in LMICs.

**Supplementary Information:**

The online version contains supplementary material available at 10.1007/s00520-026-10734-7.

## Introduction

India continues to experience a high and growing disease burden from cancer, with a prevalence rate of 100.4 per 100,000 people and cases are expected to grow by 12.8% between 2020 and 2025 [1]. New Delhi exhibited the highest cancer incidence for males and one of the highest for women in India [2], experiencing an especially pronounced increase [3]. The most common cancer sites by prevalence per 100,000 people were the digestive system (19.8), breast (15.2), genitalia (15.0), oral cavity and pharynx (13.6), and respiratory system (9.8) [1]. Among males, the most common cancer was lung cancer while for females, it was breast cancer [1].

An inherently social disease, the cancer disease burden is influenced by social determinants of health (SDHs). Among both men and women, the cancer mortality rate was over twice as high for illiterate Indians compared to those with post-secondary education [4]. Affluent patients were also found to more readily present to screening [5] and exhibit adequate disease knowledge [6]. In contrast, those in urban slums in New Delhi were found to be unaware of primary care cancer services or to identify common symptoms [7]. However, more up-to-date investigation of this issue is warranted.

There are significant costs associated with Indian cancer care with an average of 49% of household expenditure allocated towards outpatient care [8] and many relying on savings and loans from acquaintances to finance treatment [9]. Elderly patients, those utilising private care and in rural areas, typically had especially high treatment costs [10]. This greatly influenced adherence to the cancer treatment with patients who were educated to post-secondary level more likely to present to healthcare services earlier in disease progression [11] and complete treatment [12]. This phenomenon was also observed for rural and low-income patients [13]. This likely contributed to findings that palliative or advanced-stage cancer patients were from predominantly low-SES backgrounds [14].

Patients receiving treatment for cancers typically experienced discomfort from their chronic disease, such as fatigue or weakness, loss of appetite, pain or shortness of breath [15]. Management of such symptoms can be influenced by the patient’s socioeconomic background. Many patients found obstacles in coping with pain, physical discomfort and psychological distress due to their income [16]. Furthermore, a cross-sectional study from the USA found that several SDHs—age, ethnicity and social isolation—can influence symptom burden during cancer treatment [17]. No study has been identified that examined the relationship between SDHs and symptom burden among Indian palliative cancer patients using longitudinal data.

This study aims to examine the impact of SDHs—income, education and housing status—on physical, psychological and wellbeing symptoms among palliative cancer patients in New Delhi, India. It further explores the influence of SDHs at two time points in the cancer treatment continuum and symptom trajectory between the two time points. This secondary analysis utilises a real-world dataset of cancer patients receiving palliative care in New Delhi, India, to assess the role of SDHs in cancer symptom burden. Findings can inform future cancer care practices, particularly in low-resource settings and influence policy and interventions focused on improving cancer care for disadvantaged populations in India and other LMICs.

## Methods

This study is a secondary analysis of a prospective cohort dataset originally collected to validate the Integrated Palliative Outcome Scale (IPOS) in India. The study uses cross tabulation with chi-square tests and multivariate logistic regression analyses to assess the relationship between SDHs and physical, psychological and wellbeing symptom burden among the palliative cancer patients.

### Survey cohort

The IPOS dataset participants were 240 cancer patients receiving palliative care, aged 20 to 90 years, in New Delhi, India, surveyed between February 21 st and April 30th, 2021. The cohort was surveyed at two different appointments during their care continuum. Two hundred forty participants were surveyed at Time 1 (T1) during appointments between February and March. One hundred twenty participants from the T1 cohort were subsequently followed up with at Time 2 (T2) during appointments between March and April. Follow-up assessment at T2 was conducted only in a random subsample from the original IPOS validation study for the purpose of assessing test–retest reliability. Therefore, reduced numbers at T2 reflect the original study design rather than attrition. This study examined the relationship between background characteristics (income and education) and physical (pain, weakness), psychological (depression, anxiety) and wellbeing symptoms (feeling informed and resolution of personal/financial concerns [RPFC]).

Participants in the IPOS survey were asked about the following topics: background characteristics including age, gender, marital status, educational attainment, socioeconomic status (SES), income status, family structure and accommodation; clinical variables such as cancer type, specific neoplasm site as classified by the International Classification of Diseases for Oncology (ICD-O) and cancer staging according to the simplified TNM Staging System; the presence of comorbidities such as congestive heart failure, HIV, hypertension, diabetes and liver/renal/gastric disease. Patients were also asked to rank the severity of physical and psychological symptoms on a Likert scale (0 = not present at all to 4 = overwhelmingly present). General wellbeing, such as feeling informed and resolution of personal and financial concerns, was ranked on a different five-point Likert scale (0 = always, 4 = not at all).

### Variables assessed and statistical analysis

This study explored the relationship between SDHs selected from the background characteristics—income, education and slum status—and physical (pain and weakness), psychological (depression and anxiety) and wellbeing symptoms (feeling informed, resolution of personal/financial concerns [RPFC]). The SDHs are each a component of the WHO Social Determinants of Health Framework [18] while the physical and psychological symptoms were drawn from the General Symptom Distress Scale, which are common symptoms present in a variety of chronic illnesses [19]. This relationship was also assessed longitudinally through calculating symptom change scores (T2 minus T1) over the treatment course. These variables were selected as they were strong proxies for the quality of cancer care and were generalisable across cancer types.

Aside from slum status, all selected variables were collapsed from the 5-point Likert scale used in the IPOS survey into three or fewer categories to improve statistical power through reducing data sparseness. Notably, symptom severity variables were collapsed into three categories: not at all present (0.00), slightly or moderately present (1.00) and severely or overwhelmingly present (2.00). Change scores were also collapsed into three categories: deterioration (−1.00), no change (0.00), improvement (1.00). Therefore, for each variable, higher ordinal values represented more severe symptom severity. Each variable was collapsed according to proformas in Appendix 1.

Cross tabulation with chi-square analysis was utilised to identify statistically significant relationships between SDHs and symptom burden of palliative cancer patients. The full results of the cross tabulation with chi-square testing are shown in Appendix 2. Multivariate ordinal logistic regression was then utilised to assess the predictive value of SDHs. Separate models were run for each of the physical, psychological and wellbeing symptoms at both T1 and T2. Additional covariates were included: age, cancer staging and presence of any comorbidities. A binary comorbidities variable was created to indicate whether a participant reported at least one of the listed comorbid conditions: hypertension, diabetes, heart failure, HIV/AIDS or liver/renal disease.

Prior to running the model, assumptions of ordinal logistic regression were assessed. The proportional odds assumption was tested using the Test of Parallel Lines in SPSS for each ordinal logistic regression model. The assumption was satisfied for all reported models (*p* > 0.05) except one though no findings from this model were reported. Multicollinearity diagnostics were conducted by entering all predictors into a linear regression model. As shown in Table 1, variance inflation factor (VIF) values ranged from 1.009 to 1.177, with all tolerance values > 0.8, suggesting no evidence of multicollinearity.

**Table 1:** Results of multicollinearity diagnostics for all predictors used in ordinal logistic regression

Predictor	Tolerance	VIF
Age	0.849	1.177
Is this accommodation slum? Yes or no	0.938	1.067
Cancer staging	0.992	1.009
Presence of any comorbidity	0.945	1.059
Educational attainment	0.894	1.119
Income	0.878	1.139

The assumption of linearity of the logit for the continuous predictor—age measured in years—was assessed using the Box–Tidwell test, where a non-significant interaction (*p* > 0.05) indicated the assumption was satisfied. The linearity of the logit assumption was met for age in all models (all *p*-values > 0.05). Therefore, all assumptions of ordinal logistic regression were satisfied, permitting its use in this analysis. The complete results of the multivariate, ordinal logistic regression with all covariates and associated Test of Parallel Lines are in Appendix 3.

## Results

### Cohort characteristics

The background characteristics of participants using original, non-collapsed variables of the survey cohort are represented in Table 2. The cohort patients had a wide age range, though most (49.6%) were between 40 and 59 years old. Nearly one in five were aged 20–39 (18.3%), while older adults aged 60–74 made up 23.4% of the sample, and just 8.8% were 75 or older. The sample was predominantly female (69.6%). Educational attainment was varied, with a notable proportion (27.5%) reporting no formal education or being illiterate. Only 15% had university-level education (10.8% with a Bachelor’s, 4.2% with a Master’s), highlighting a generally low-education population. This was contrary to Delhi’s population, which had a literacy rate of 89.4% [20]. Only 8.8% of the cohort were living in slum accommodation and a majority, compared to 13% of New Delhi’s population [21]. Baseline comparison between all participants and those completing T2 showed no significant differences in education (*p* = 0.294), income (*p* = 0.758), or slum residence (*p* = 0.276), suggesting limited evidence of attrition bias.

**Table 2:** Background characteristics of participants (*n* = 240)

Variable	Category	*n* (%)
Age	20–39	44 (18.3)
	40–59	119 (49.6)
	60–74	56 (23.4)
	75 +	21 (8.8)
Gender	Female	167 (69.6)
	Male	73 (30.4)
Education qualification	No formal education or illiterate	66 (27.5)
	Primary	56 (23.3)
	Secondary	59 (24.6)
	Senior Secondary	22 (9.2)
	Graduate (Bachelor’s degree)	26 (10.8)
	Postgraduate (Master’s degree)	10 (4.2)
	Missing	1 (0.4)
Income status	Living comfortably on present income	30 (12.5)
	Coping on present income	58 (24.2)
	Difficult on present income	89 (37.1)
	Very difficult on present income	57 (23.8)
	Missing	6 (2.5)
Accommodation details	Government or company owned	11 (4.6)
	Their own	161 (67.1)
	Rented	62 (25.8)
	Charitable shelter	3 (1.3)
	Missing	3 (1.3)
Slum status	Yes	21 (8.8)
	No	219 (91.3)
Cancer staging	Unknown or N/A	34 (14.2)
	Stage 1	12 (5.0)
	Stage 2	66 (27.5)
	Stage 3	59 (24.6)
	Stage 4	67 (27.9)
	Missing	2 (0.8)
Cancer topography (ICD-O Classification)	C00–C14 Lip, oral cavity and pharynx	55 (22.9)
	C15–C26 Digestive organs	31 (12.9)
	C30–C39 Respiratory system and intrathoracic organs	13 (5.4)
	C40–C41 Bones, joints and articular cartilage	2 (0.8)
	C42 Hematopoietic and reticuloendothelial systems	15 (6.3)
	C44 Skin	1 (0.4)
	C48 Retroperitoneum and peritoneum	1 (0.4)
	C49 Connective, subcutaneous and other soft tissues	2 (0.8)
	C50 Breast	67 (27.9)
	C51–C58 Female genital organs	39 (16.3)
	C64–C68 Urinary tract	3 (1.3)
	C69–C72 Eye, brain and other parts of central nervous system	5 (2.1)
	C73–C75 Thyroid and other endocrine glands	3 (1.3)
	C80 Unknown primary site	1 (0.4)
	Missing	2 (0.8)
Any comorbidities	Yes	42 (18.0)
	No	196 (82.0)
	Missing	2 (0.8)

A majority of patients reported financial difficulty: 37.1% found it difficult and 23.8% very difficult to live on their present income. India’s urban poverty rate using the World Bank’s LMIC poverty line was 17.2% [22]. Cancer staging was relatively evenly spread between Stage 2 (27.5%), Stage 3 (24.6%), and Stage 4 (27.9%), with 14.2% having unknown or unreported stage. The most common cancer diagnoses were cancers of the breast (27.9%), lip, oral cavity and pharynx (22.9%), female genital organs (16.3%) and digestive organs (12.9%), with all remaining categories under 7%. Eighteen percent reported comorbidities, such as congestive heart failure, diabetes or HIV/AIDS. This dataset thus reflects a largely low-income, low educational attainment population relative to Delhi’s population with a heavy burden of advanced-stage disease and cancers common among females.

**Fig. 1:**
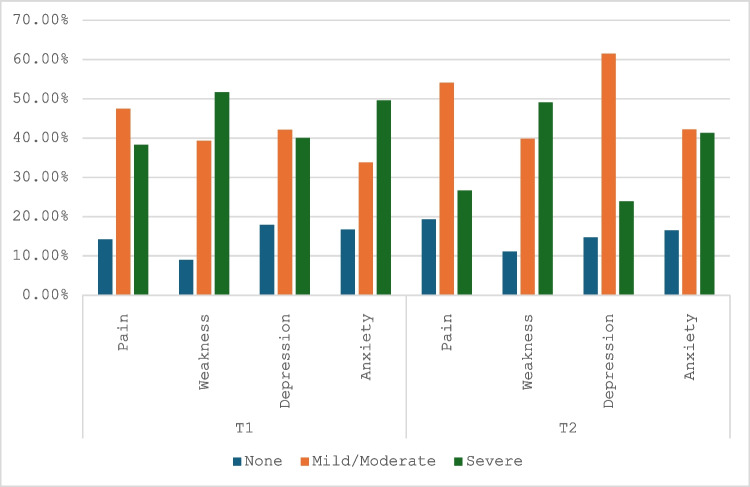
Distribution of physical and psychological symptom severity at T1 and T2

Figure 1 shows the distribution of symptom severity at T1 and T2 using variables collapsed according to proformas in Appendix 1. At T1, the majority of participants reported mild to moderate symptom burden across physical and psychological domains. Pain was present in an overwhelming majority of participants, with 47.5% reporting mild/moderate pain and 38.3% reporting severe pain. A similar trend was observed for weakness, where 51.3% experienced it severely and 36.7% to a mild/moderate degree. Psychological symptoms were also prevalent, with 49.2% of patients experiencing mild/moderate anxiety and 38.3% reporting it as severe. Depression appeared slightly less severe, with 41.3% mild/moderate and 37.1% severe. At T2, mild/moderate pain increased to 52.5%, and mild/moderate depression rose to 58.3%, suggesting some decline in symptom severity in these domains. In contrast, severe anxiety and weakness remained persistently high at 39.2% and 47.5%, respectively. These findings suggest that some physical and psychological symptoms may improve during the treatment continuum while others persist highlighting the need for analysis of predictors of symptom trajectory.

### Relationship between SDHs and symptom severity

Several statistically significant results were observed from chi-square analysis. At T1, there was a significant association between education and feeling informed (*X*^2^(4) = 11.100, *p* = 0.025) with a higher proportion of patients with a post-secondary education reporting that they felt more adequately informed about their care relative to those without formal education. Coping on income demonstrated a number of strong associations with feeling less severe weakness (*X*^2^(4) = 16.254, *p* < 0.001), more adequately informed (*X*^2^(4) = 7.986, *p* = 0.018) and less severe anxiety (*X*^2^(4) = 7.333, *p* = 0.026) at T1. Living in slum accommodation was associated with weakness (*X*^2^(2) = 6.047, *p* = 0.049) and depression (*X*^2^(2) = 6.971, *p* = 0.031) with those in slums more likely to report moderate or severe weakness and severe depression.

At T2, there were additional significant findings. Education was associated with less severe pain (*X*^2^(4) = 14.459, *p* = 0.006) and weakness (*X*^2^(4) = 15.423, *p* = 0.004) while coping on income was likewise associated with less severe pain (*X*^2^(4) = 6.537, *p* = 0.038) and lower anxiety (*X*^2^(4) = 7.856, *p* = 0.020). Exploratory trends were observed between coping on income and depression (*X*^2^(4) = 4.903, *p* = 0.086) and pain severity (*X*^2^(4) = 5.136, *p* = 0.077) at T1, as well as RPFC at T2 (*X*^2^(4) = 4.880, *p* = 0.087), although neither reached statistical significance.

In the ordinal logistic regression analysis, both education and income were significantly associated with weakness at T1. Compared to those with tertiary education, those with no education had 2.84 times higher odds of reporting more severe weakness at T1 (OR = 2.84, 95% CI = 1.25–6.42, *p* = 0.012), and those with secondary had 2.28 times higher odds (OR = 2.28, 95% CI = 1.00–5.21, *p* = 0.050). Similarly, compared to those coping on their income, individuals not coping on their income had 2.67 times higher odds of reporting more severe weakness at T1 (OR = 2.67, 95% CI = 1.48–4.82, *p* = 0.001) and more severe pain at T2 (OR = 2.44, 95% CI 1.02–5.81, *p* = 0.044).

Income likewise demonstrated a significant association with depression at T1 with those not coping on their current income having 1.94 times higher odds of reporting more severe depression (OR = 1.94, 95% CI 1.10–3.40, *p* = 0.021). Furthermore, those not resident in slum accommodation were 75% less likely to experience severe depression at T1 (OR = 0.25, 95% CI 0.09–0.70, *p* = 0.008). At T2, not coping on income was associated with 4.50 times higher odds of no RPFC (OR = 4.50, 95% CI 1.00–20.25, *p* = 0.050) and 2.58 times higher odds of feeling less informed about care (OR = 2.58, 95% CI 1.03–6.44, *p* = 0.043). Exploratory trends were observed between income and pain (*p* = 0.101) and anxiety (*p* = 0.078) at T1 though these did not reach statistical significance.

### Relationship between SDHs and symptom severity changes between T1 and T2

Fewer significant associations were found between background characteristics and changes in symptom severity between T1 and T2. Cross tabulation with chi-square tests demonstrated an association between education and a change in weakness severity between T1 and T2 (*X*^2^(4) = 9.884, *p* = 0.042) with those with tertiary education being less likely to deteriorate than those with no formal or secondary education. There were no associations found between income or slum status and any symptom severity changes between T1 and T2. All significant findings from the cross tabulation with chi-square are shown in Table 3.

**Table 3:** Significant associations between socioeconomic determinants and symptom outcomes (cross tabulation with chi-square test)

Outcome	Predictor	*χ*^2^ (df)	*p*-value	Association summary
Feeling informed T1	Education	11.100 (4)	0.025	Post-secondary education → more likely to feel informed
Weakness T1	Income	16.254 (4)	< 0.001	Coping income → less severe weakness
Feeling informed T1	Income	7.986 (4)	0.018	Coping income → more likely to feel informed
Anxiety T1	Income	7.333 (4)	0.026	Coping income → less severe anxiety
Weakness T1	Slum residence	6.047 (2)	0.049	Slum residents → more likely to report moderate/severe weakness
Depression T1	Slum residence	6.971 (2)	0.031	Slum residents → more likely to report severe depression
Pain T2	Education	14.459 (4)	0.006	Higher education → less severe pain
Weakness T2	Education	15.423 (4)	0.004	Higher education → less severe weakness
Pain T2	Income	6.537 (4)	0.038	Coping income → less severe pain
Anxiety T2	Income	7.856 (4)	0.020	Coping income → less severe anxiety
Change in weakness (T1–T2)	Education	9.884 (4)	0.042	Tertiary education → less likely to deteriorate in weakness

In the ordinal logistic regression analysis, there were some significant associations found between SDHs and changes in symptoms between T1 and T2. Patients with a secondary education had lower odds of improvement in weakness between T1 and T2 relative to those with tertiary education (OR = 0.18, 95% CI 0.041–0.769, *p* = 0.021). Unexpectedly, those living in a non-slum residence exhibited lower odds of an improvement in pain symptoms (OR = 0.16, 95% CI 0.038–0.657, *p* = 0.011) while those not coping on their income had 3.5 times higher odds of showing greater RPFC between T1 and T2 compared to those coping on their income (OR = 3.51, 95% CI 1.11–11.09, *p* = 0.032). These findings were more likely to reflect regression to the mean than genuine clinical improvement as discussed below. Lastly, there was a trend towards significance between income and changes in weakness with those not coping showing lower odds of improvement, although this did not reach statistical significance (OR = 0.44, 95% CI 0.18–1.10, *p* = 0.079). All significant findings from the multivariate, ordinal logistic regression analysis are displayed in Table 4.

**Table 4:** Significant associations between socioeconomic determinants and symptom outcomes (multivariate logistic regression)

Outcome	Predictor	OR	95% CI	*p*-value
Weakness T1	Income (not coping)	2.67	1.48–4.82	0.001
	Education (none/primary)	2.84	1.25–6.42	0.012
	Education (secondary)	2.28	1.00–5.21	0.050
Pain T2	Income (not coping)	2.44	1.02–5.81	0.044
Depression T1	Income (not coping)	1.94	1.10–3.40	0.021
	Slum (not resident)	0.25	0.09–0.70	0.008
RPFC T2	Income (not coping)	4.50	1.00–20.25	0.050
Informed T2	Income (not coping)	2.58	1.03–6.44	0.043
Change weakness	Education (secondary)	0.18	0.041–0.769	0.021
Change pain	Slum (not resident)	0.16	0.038–0.657	0.011
Change RPFC	Income (not coping)	3.51	1.11–11.09	0.032

## Discussion

This study demonstrates that SDHs—education, income, and housing—are strongly associated with cancer symptom burden among this survey cohort. The most consistent findings were observed for education and coping on income, both of which influenced patients’ reported weakness, pain, anxiety, depression and feeling informed about the cancer care continuum.

Higher educational attainment was associated with lower severity of weakness and pain and with feeling more informed about care. This was consistent with education being a predictor of greater pain and lesser quality of life among cancer patients in the USA [23] and of impaired physical function in Denmark [24]. In an LMIC context, low-SES was similarly associated with greater chronic pain and psychological suffering across Sri Lanka, China, India, Vietnam and Myanmar [25]. However, longitudinal analyses revealed that patients with secondary education were less likely to observe improvements in weakness compared to those with tertiary education. This gradient suggests that while education broadly confers advantages, tertiary education specifically provides the strongest protection.

The patient coping on its present income was another key determinant. Patients reporting difficulty coping were more likely to report worse weakness, pain, anxiety and depression, and less likely to feel informed or report RPFC at follow-up. There are several potential mechanisms that explain this association. Firstly, affluent patients are more likely to utilise private healthcare facilities, which observe better healthcare outcomes than public facilities [26]. Informal payments are also widespread in the Indian healthcare infrastructure, with almost three-quarters of pregnant women making such payments during their maternity care [27]. This phenomenon may also be present in cancer care, allowing more wealthy patients to finance superior care. However, more research on the prevalence of informal payments in cancer care in India is warranted. Lastly, income is inextricably linked with India’s caste system [28]. Low caste patients had poorer access to healthcare services in India due to discrimination and stigma [29], potentially contributing to the higher symptom burden among low-SES palliative cancer patients.

Housing conditions also played an important role. Patients residing in slum accommodation were more likely to report weakness and severe depression, and regression analyses indicated that those not living in slums had significantly lower odds of depression at T1. This is consistent with studies indicating that slum residents in New Delhi have poorer knowledge of cancer symptoms [7] and reduced accessibility to healthcare services [30].

However, there were some surprising caveats to the relationship between low-SES indicators and greater symptom burden. Two unexpected findings include that patients not coping on their income were more likely to report improvement in RPFC over time and that slum residents were more likely to report improvements in pain over time. These were both unexpected findings and contradictory to the model’s other results, which demonstrated a strong general association between non-slum residence and coping on income with a reduced symptom burden. The most likely explanation for the paradoxical findings is regression to the mean: patients with the most severe baseline symptoms, such as pronounced personal/financial concerns, naturally tend to report greater improvement over time, even in the absence of substantive change [31]. Furthermore, the phenomenon of response shift—where patients recalibrate how they interpret symptom severity or resolution—could lead individuals who initially report intense struggle to perceive marginal progress as especially meaningful, compared to those with milder complaints at baseline [32, 33].

There is another possible, though less probable, explanation for these paradoxical findings. Those coping on their income were already more likely to have at least partially resolved personal and financial concerns at T1 with 10.2% of coping patients having complete RPFC compared to only 5.6% of non-coping patients, leaving more potential for improvement amongst the non-coping group. However, similar proportions—14.2% versus 14.3%—of non-slum and slum residents reported no pain at T1, meaning this would not explain slum residents experiencing greater improvement in pain symptoms between T1 and T2.

These findings underscore the importance of integrating social and economic considerations into cancer care delivery. Greater emphasis should be placed on mental health care for cancer patients, particularly given the elevated psychological burden observed among low-SES groups and evidence that improved mental health care can enhance prognosis [34]. Consistent with our findings, low-SES patients typically reported more severe symptoms at baseline, potentially reflecting delayed presentation to healthcare services [35]. Interventions focused on patient education, early detection, and timely presentation should therefore be developed and implemented in low-SES communities such as slums. Although such approaches have shown promise in LMICs, they require more standardised evaluation [36]. Therefore, patient education should be complemented by reforms, such as income support, which has been found to ease financial insecurity and improve treatment adherence [37].

There are some notable limitations to this paper and the IPOS dataset utilised, which future studies should aim to address. Firstly, there are limits to the longitudinal analysis component of this study as the IPOS survey only recorded results at two separate stamps approximately 1 month apart. Furthermore, only half of the cohort took part in the survey at the second timestamp. Secondly, while ordinal change scores allowed a longitudinal comparison, simplifying these into only three categories (deterioration, no change or improvement) may have obscured nuance in symptom progression. Thirdly, as a convenience sample conducted only in New Delhi, it has limited generalisability to India overall as it only surveyed 240 patients. Participants had comparatively low educational attainment and SES relative to New Delhi overall potentially limiting generalisability to the city’s population. Finally, this study controlled for patient comorbidities using a binary variable. Future studies should attempt to account for the severity of comorbidities and, therefore, their relative impact on cancer prognosis and explore the mechanisms underlying paradoxical associations, particularly the apparent symptom improvements among financially strained patients.

## Conclusion

This analysis demonstrates that SDHs—income, education and housing—are significantly associated with the physical, psychological and wellbeing symptom burden of palliative cancer patients in New Delhi, India. Patients with lower education and financial insecurity consistently reported more severe pain, weakness, depression, anxiety and poorer perceptions of being informed, while slum residence further compounded psychological vulnerability. These disparities were evident not only at baseline but also at follow-up. Although paradoxical associations, such as greater improvement in financial concerns among low-income patients, may reflect regression to the mean or reporting bias, the broader pattern indicates that inequities are rooted in systemic disadvantages in access to and quality of care. These findings highlight the urgent need to integrate social and economic considerations into cancer care delivery, including mental health support, patient education, early detection interventions and financial protection mechanisms tailored to low-SES populations. Addressing these inequities is critical to improving the cancer care experience in India and other LMICs, where the most disadvantaged patients continue to bear the heaviest cancer disease burden.

## Supplementary Information

Below is the link to the electronic supplementary material.

ESM 1 Supplementary Material 1 (PDF 80.2 KB)

ESM 2 Supplementary Material 2 (XLSX 70.8 KB)

## Data Availability

The IPOS survey data is available on request. The complete findings of this study's cross tabulation with Chi Square and multivariate logistic regression analysis are attached as supplemental material.
