# Supplementary material for: Influence of socioeconomic status on physical and psychological wellbeing during the cancer care continuum: a longitudinal and logistic regression analysis of cancer patients in New Delhi, India
Source: Support Care Cancer. 2026 May 7;34(6):507. doi: 10.1007/s00520-026-10734-7 (PMC13149577; doi:10.1007/s00520-026-10734-7)
Supplement: Supplementary file 1 — Supplementary Material 1 (PDF 80.2 KB) [file 520_2026_10734_MOESM1_ESM.pdf]

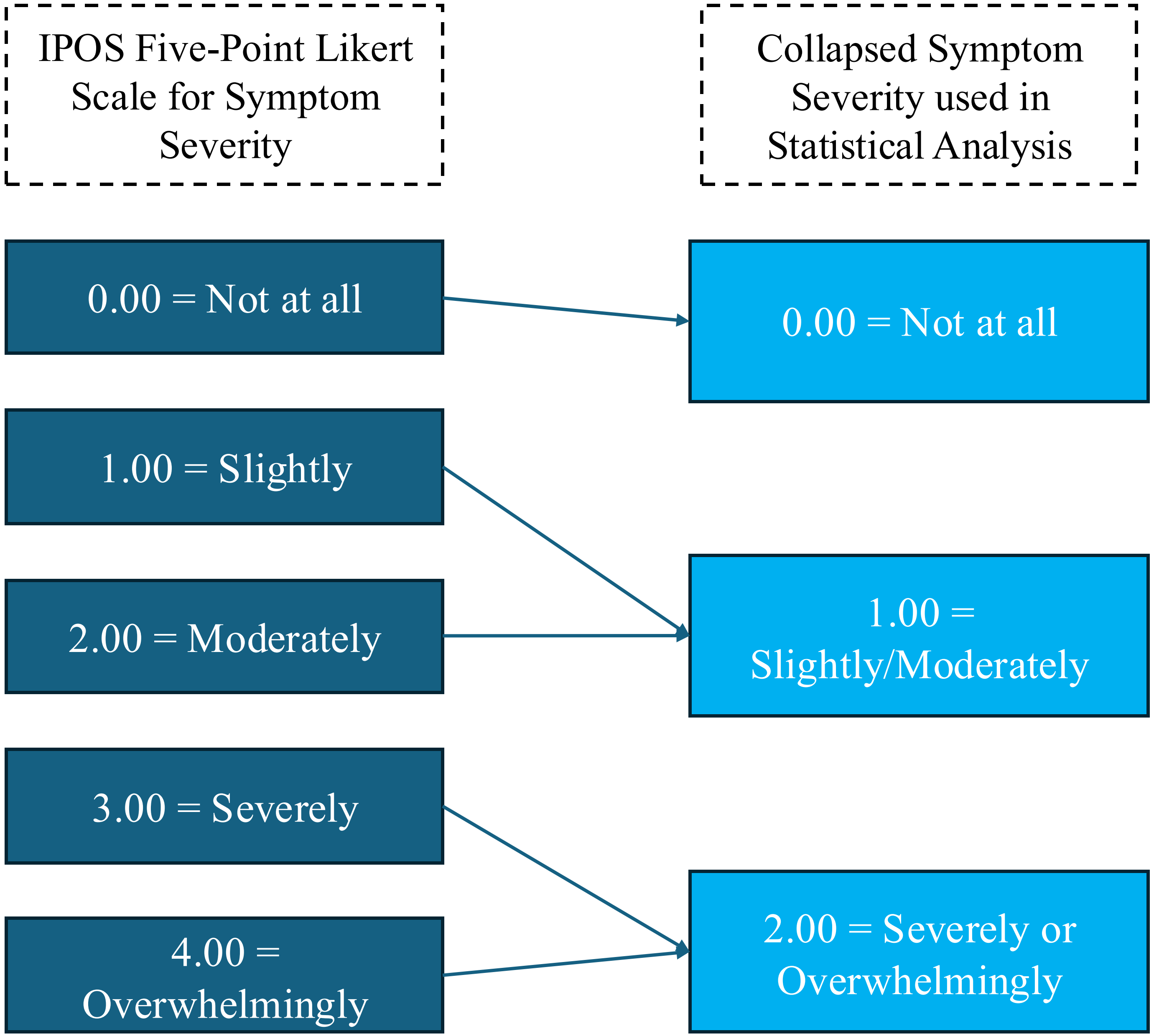
IPOS Five-Point Likert Scale for Symptom Severity
Collapsed Symptom Severity used in Statistical Analysis
0.00 = Not at all
1.00 = Slightly
2.00 = Moderately
3.00 = Severely
4.00 = Overwhelmingly
0.00 = Not at all
1.00 = Slightly/Moderately
2.00 = Severely or Overwhelmingly

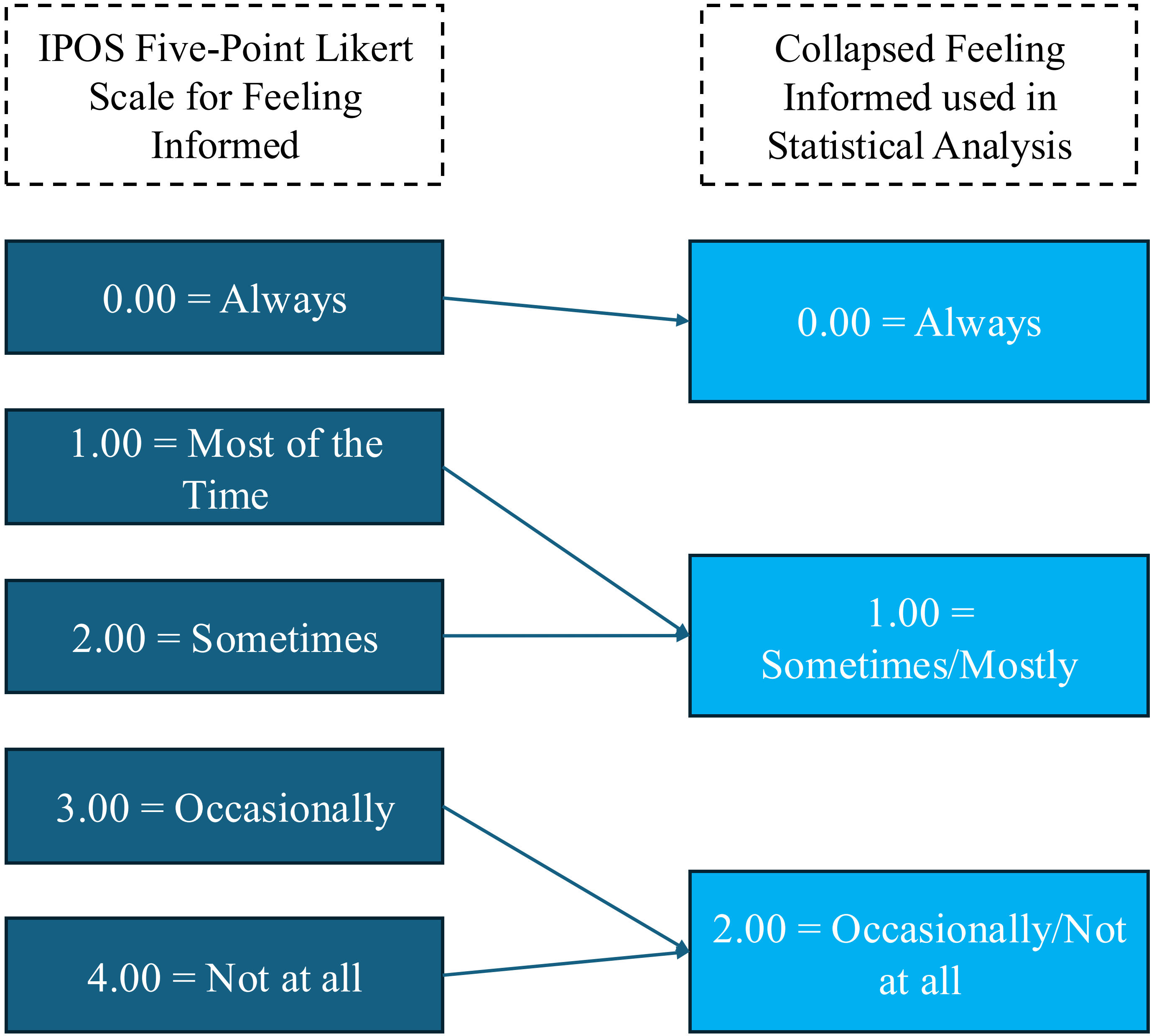
IPOS Five-Point Likert Scale for Feeling Informed
Collapsed Feeling Informed used in Statistical Analysis
0.00 = Always
1.00 = Most of the Time
2.00 = Sometimes
3.00 = Occasionally
4.00 = Not at all
0.00 = Always
1.00 = Sometimes/Mostly
2.00 = Occasionally/Not at all

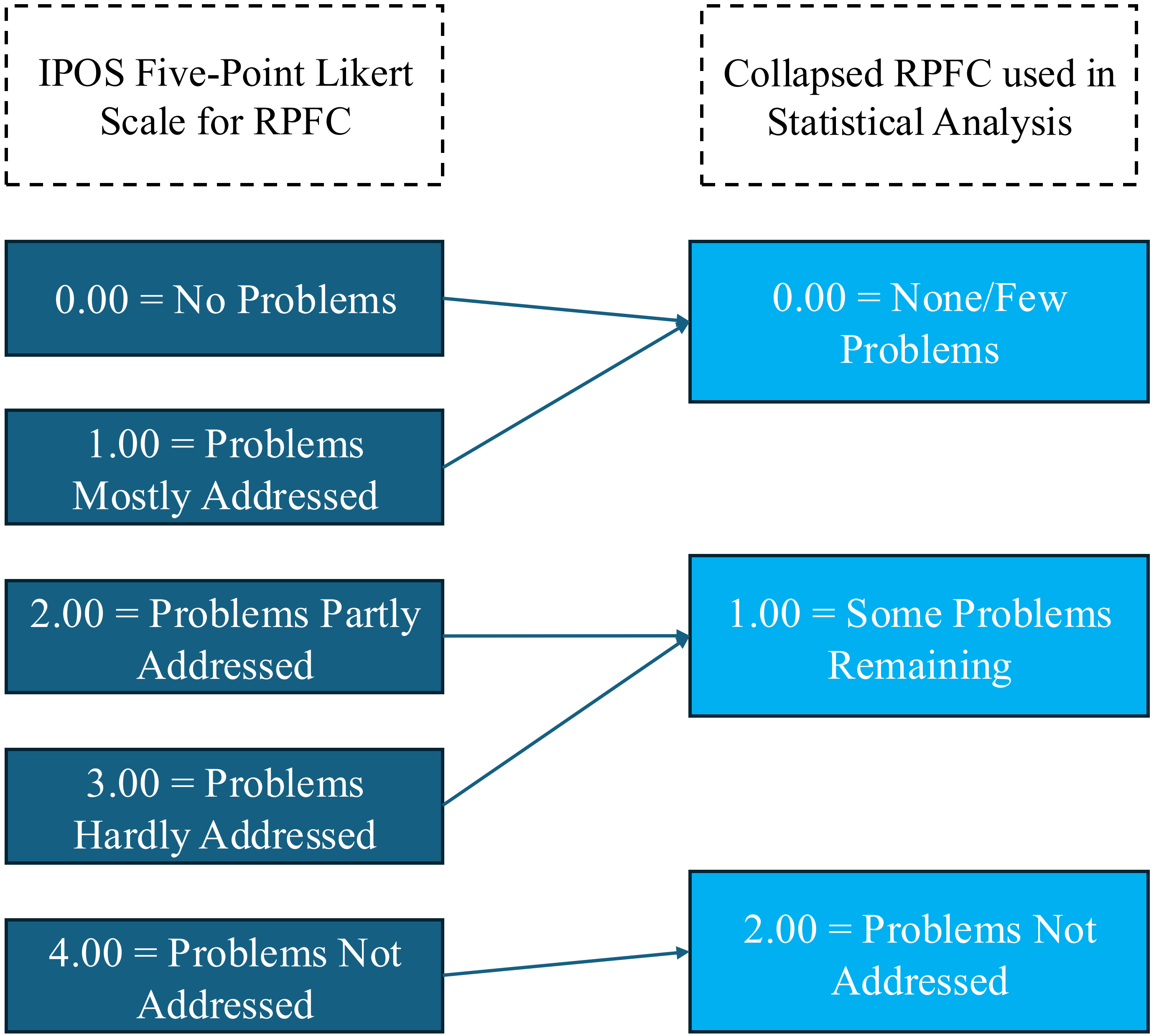
IPOS Five-Point Likert Scale for RPFC
Collapsed RPFC used in Statistical Analysis
0.00 = No Problems
1.00 = Problems Mostly Addressed
2.00 = Problems Partly Addressed
3.00 = Problems Hardly Addressed
4.00 = Problems Not Addressed
0.00 = None/Few Problems
1.00 = Some Problems Remaining
2.00 = Problems Not Addressed

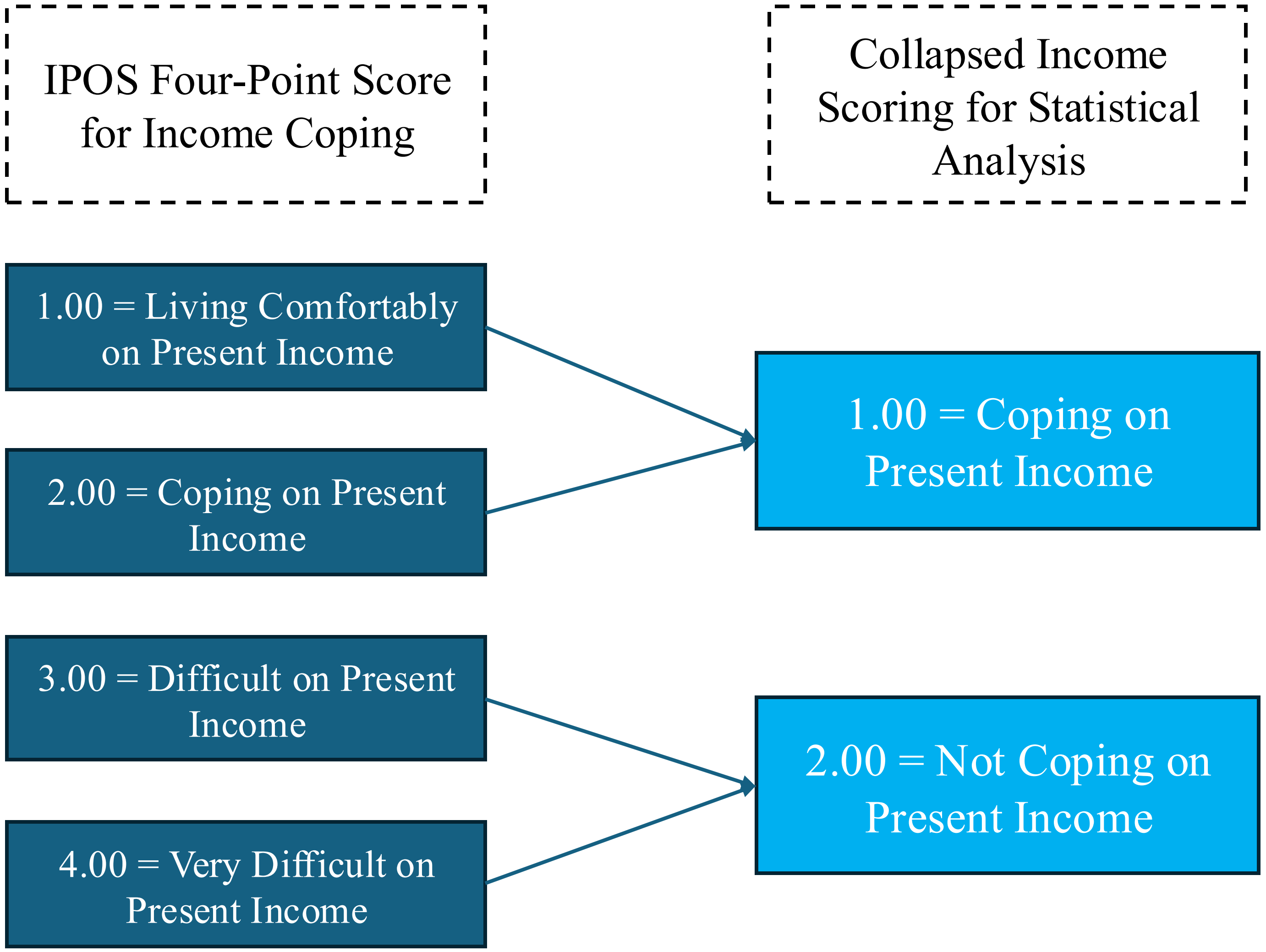
IPOS Four-Point Score for Income Coping
Collapsed Income Scoring for Statistical Analysis
1.00 = Living Comfortably on Present Income
2.00 = Coping on Present Income
3.00 = Difficult on Present Income
4.00 = Very Difficult on Present Income
1.00 = Coping on Present Income
2.00 = Not Coping on Present Income

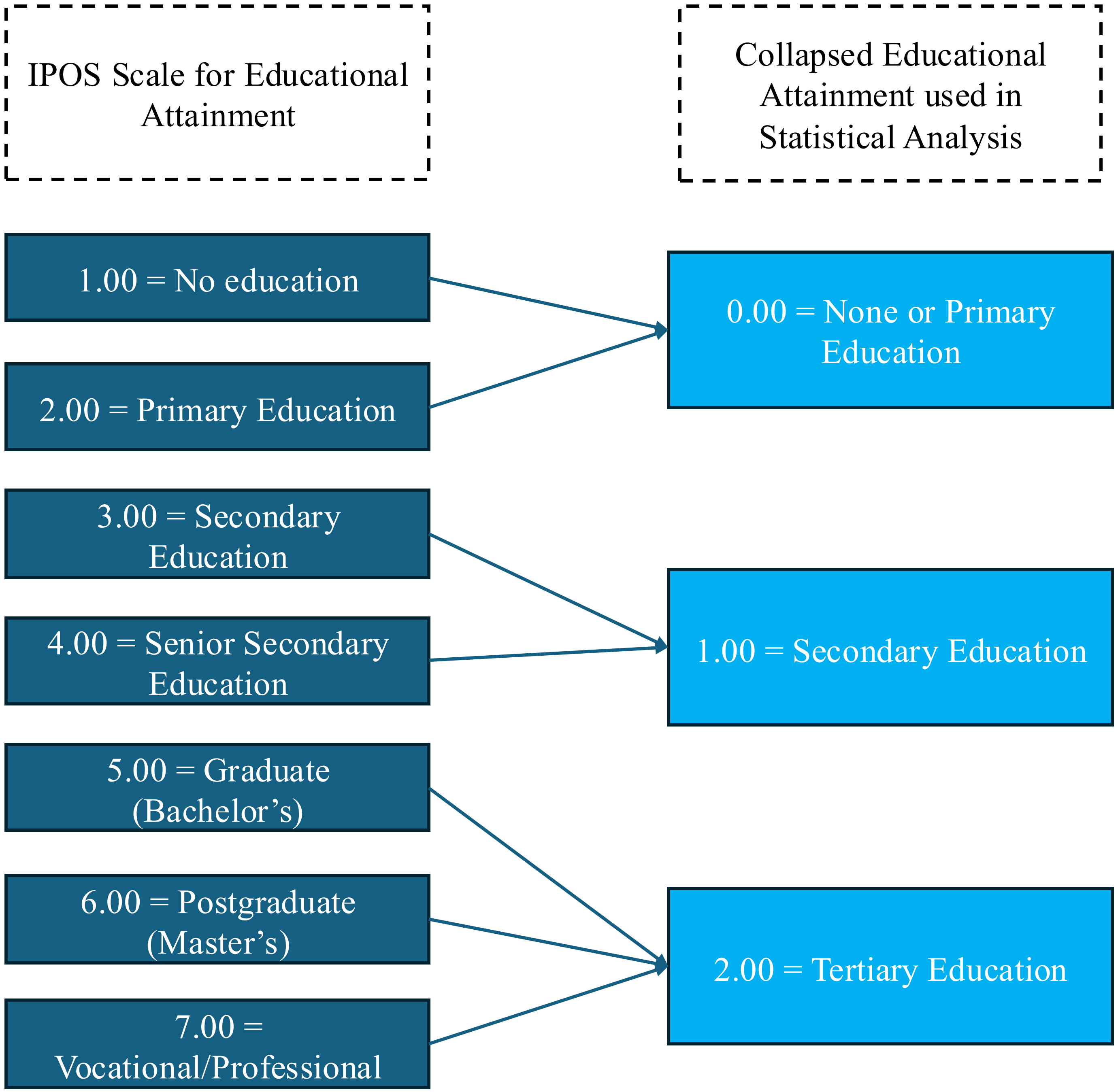
IPOS Scale for Educational Attainment
Collapsed Educational Attainment used in Statistical Analysis
1.00 = No education
2.00 = Primary Education
3.00 = Secondary Education
4.00 = Senior Secondary Education
5.00 = Graduate (Bachelor's)
6.00 = Postgraduate (Master's)
7.00 = Vocational/Professional
0.00 = None or Primary Education
1.00 = Secondary Education
2.00 = Tertiary Education

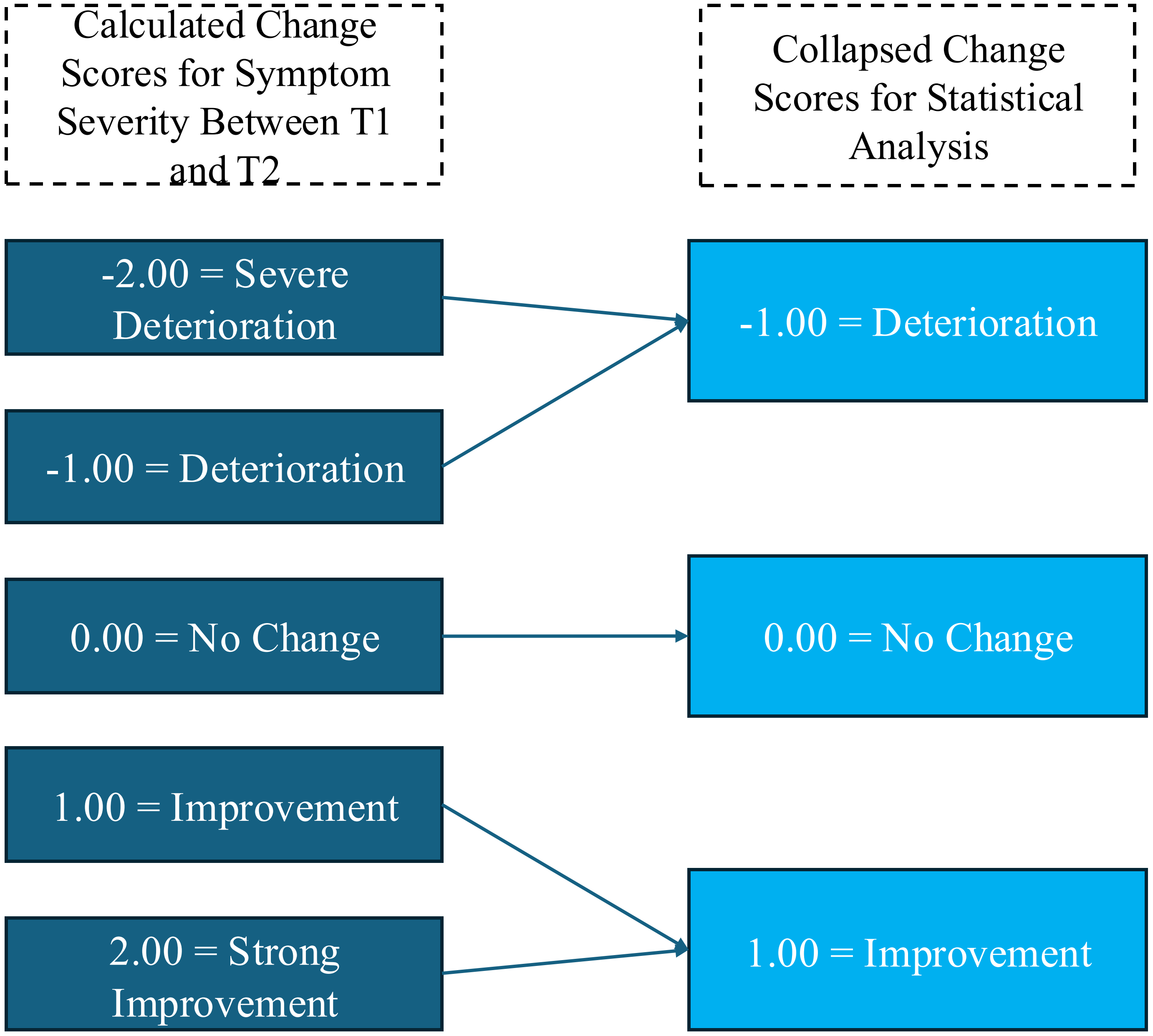
Calculated Change Scores for Symptom Severity Between T1 and T2
Collapsed Change Scores for Statistical Analysis
-2.00 = Severe Deterioration
-1.00 = Deterioration
0.00 = No Change
1.00 = Improvement
2.00 = Strong Improvement
-1.00 = Deterioration
0.00 = No Change
1.00 = Improvement
